# Relaxin family peptide receptor 3 (RXFP3) expressing cells in the zona incerta/lateral hypothalamus augment behavioural arousal

**DOI:** 10.1111/jnc.16217

**Published:** 2024-09-04

**Authors:** Brandon K. Richards, Sarah S. Ch'ng, Ariel B. Simon, Terence Y. Pang, Jee Hyun Kim, Andrew J. Lawrence, Christina J. Perry

**Affiliations:** ^1^ The Florey Institute of Neuroscience and Mental Health Parkville Victoria Australia; ^2^ Florey Department of Neuroscience and Mental Health The University of Melbourne Parkville Victoria Australia; ^3^ School of Psychological Sciences Macquarie University North Ryde New South Wales Australia; ^4^ Institute of Health and Sports (IHES) Victoria University Footscray Victoria Australia; ^5^ IMPACT—The Institute for Mental and Physical Health and Clinical Translation, School of Medicine, Deakin University Geelong Victoria Australia

**Keywords:** arousal, defensive response, fear conditioning, hypothalamus, RXFP3, zona incerta

## Abstract

Fear‐related psychopathologies, such as post‐traumatic stress disorder, are linked to dysfunction in neural circuits that govern fear memory and arousal. The lateral hypothalamus (LH) and zona incerta (ZI) regulate fear, but our understanding of the precise neural circuits and cell types involved remains limited. Here, we examined the role of relaxin family peptide receptor 3 (RXFP3) expressing cells in the LH/ZI in conditioned fear expression and general arousal in male RXFP3‐Cre mice. We found that LH/ZI RXFP3+ (LH/ZI^RXFP3^) cells projected strongly to fear learning, stress, and arousal centres, notably, the periaqueductal grey, lateral habenula, and nucleus reuniens. These cells do not express hypocretin/orexin or melanin‐concentrating hormone but display putative efferent connectivity with LH hypocretin/orexin+ neurons and dopaminergic A13 cells. Following Pavlovian fear conditioning, chemogenetically activating LH/ZI^RXFP3^ cells reduced fear expression (freezing) overall but also induced jumping behaviour and increased locomotor activity. Therefore, the decreased freezing was more likely to reflect enhanced arousal rather than reduced fear. Indeed, stimulating these cells produced distinct patterns of coactivation between several motor, stress, and arousal regions, as measured by Fos expression. These results suggest that activating LH/ZI^RXFP3^ cells generates brain‐wide activation patterns that augment behavioural arousal.

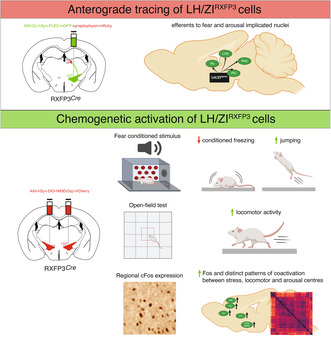

## Graphical abstract

Relaxin family peptide receptor 3 (RXFP3) is an inhibitory G‐protein coupled receptor expressed widely throughout the mammalian brain. We show that RXFP3‐expressing cells in the mouse zona incerta and lateral hypothalamus (LH/ZI^RXFP3^) send efferent projections to fear and arousal‐implicated nuclei. Following Pavlovian fear conditioning, chemogenetically activating LH/ZI^RXFP3^ cells reduced conditioned freezing but induced jumping behaviour in a subset of mice. Additionally, chemogenetically activating these cells increased general locomotor activity and Fos activation in stress, motor, and arousal nuclei. Overall, our findings provide neuroanatomical and functional evidence that LH/ZI^RXFP3^ cells participate in behavioural arousal. (Created with BioRender.com).

**Abbreviations**
AEC: Animal Ethics Committee
ANOVA: analysis of variance
BAC: bacterial artificial chromosome
CNO: clozapine‐N‐oxide
CS: conditioned stimulus
DAB: 3,3′‐Diaminobenzidine tetrahydrochloride
DREADD: designer receptor exclusively activated by designer drug
IHC: immunohistochemistry
LH: lateral hypothalamus
mGFP: membrane‐bound green fluorescent protein
NI: nucleus incertus
RM: repeated measures
RRID: Research Resource Identifier
RXFP3: relaxin family peptide 3 receptor
US: unconditioned stimulus
ZI: zona incerta
ZId: zona incerta, dorsal
ZIr: zona incerta, rostral
ZIv: zona incerta, ventral

## 1 INTRODUCTION

Relaxin family peptide receptor 3 (RXFP3) is a ligand‐activated G_i/o_ protein‐coupled receptor and the cognate receptor for relaxin‐3 (Bathgate et al., 2006), a conserved neuropeptide primarily synthesised in GABAergic nucleus incertus (NI) neurons (Burazin et al., 2002; Ma et al., 2007). The soma of relaxin‐3+ neurons is found almost exclusively in the NI and, from here, project throughout the brain to numerous RXFP3‐expressing regions involved in arousal, stress, cognition, and motivation (Ch'ng et al., 2019; Ma et al., 2017; Smith et al., 2010). Relaxin‐3/RXFP3 signalling regulates alcohol‐seeking (Ryan et al., 2013; Shirahase et al., 2016; Walker et al., 2017), feeding (de Ávila et al., 2018, 2020; DeChristopher et al., 2019), anxiety‐like behaviour (de Ávila et al., 2020; Marwari et al., 2019; Zhang et al., 2015), and spatial learning (Albert‐Gascó et al., 2017; Haidar et al., 2017, 2019). However, few studies have explored how particular RXFP3‐expressing regions contribute to these functions.

One such region is the zona incerta (ZI; Smith et al., 2010), an inhibitory subthalamic nucleus suggested to be an integrative node for numerous behaviours, including sleep, locomotion, motivation, and defensive behaviour (Wang et al., 2019). A growing body of evidence implicates the ZI in conditioned fear expression. However, many studies have reported conflicting results, so the precise role of the ZI remains unresolved. Optically activating GABAergic ZI neurons reduced conditioned fear expression and improved extinction memory recall, whereas inhibiting these cells enhanced fear expression and impaired extinction acquisition (Chou et al., 2018; Venkataraman et al., 2019). These findings suggest that these cells are required to inhibit fear memory. However, inactivating central amygdala inputs to ZI parvalbumin+ neurons impaired fear learning and long‐term fear memory retrieval (Zhou et al., 2018), suggesting that this projection is necessary to acquire and store fear memory.

Given the known neuronal heterogeneity of the ZI (Mitrofanis, 2005), examining other neurochemically defined subpopulations is necessary to parse out a more nuanced role of the ZI in conditioned fear expression. Therefore, the ZI's role in conditioned fear expression may be further elucidated by examining ZI RXFP3+ cells. This is warranted as the topographical organisation of the relaxin‐3/RXFP3 system also overlaps substantially with circuits involved in learning, memory, and stress (Ma et al., 2007; Smith et al., 2010). Additionally, relaxin‐3/RXFP3 signalling promotes behavioural activation (Ryan et al., 2011) associated with an increased arousal state (Smith et al., 2012, 2013). Indeed, changes in arousal levels can regulate the effectiveness of fear extinction recall (Giustino & Maren, 2018), and dysregulation of the arousal system contributes to the pathophysiology of many fear‐related disorders, such as post‐traumatic stress disorder (Bierwirth & Stockhorst, 2022). Given that it has also been suggested that the ZI processes interoceptive signals such as arousal state to gate behavioural outputs (Trageser et al., 2006; Trageser & Keller, 2004), we hypothesised that ZI RXFP3+ cells would be involved in regulating conditioned fear expression.

In the present study, we examined the phenotype and efferent connectivity of ZI RXFP3+ neurons to confirm their neuroanatomical congruency with known circuits involved in conditioned fear expression and behavioural arousal. Subsequently, we functionally interrogated these neurons using a Pavlovian fear conditioning paradigm and assessed the effects of activating these cells on brain‐wide activation patterns.

## 2 METHODS

### 2.1 Animals

All studies were conducted in accordance with the Prevention of Cruelty to Animals Act (2004), under the guidelines of the National Health and Medical Research Council Code of Practice for the Care and Use of Animals for Experimental Purposes in Australia (2013) and approved by the Animal Ethics Committee (AEC) at the Florey Institute of Neuroscience and Mental Health (AEC number: 17‐030). A total of 111 mice were used. Inbred adult male RXFP3‐Cre mice (*n* = 107; Ch'ng et al., 2019) were used in all experiments except for cell phenotyping; here, RXFP3‐Cre mice were crossed with Rosa26eYFP mice (obtained from Dr Tobias Merson, while at Monash University) to generate RXFP3‐Cre‐eYFP reporter mice (*n* = 4). Mice were group‐housed (2–6 per cage) until surgery and maintained on a 12‐h light–dark cycle (lights on at 7 am) in a temperature‐controlled environment (21°C – 23°C) with nesting material and ad libitum access to standard chow (Ridley Agriproducts, Melbourne, Australia) and water. Following stereotaxic surgery, mice were single‐housed but otherwise maintained under the same conditions. Of the 107 RXFP3‐Cre mice, 41 were excluded from the final analysis for the following reasons: virus placement unable to be visualised (hM3Dq virus *n* = 19; mCherry control virus *n* = 17), missed viral injection (hM3Dq virus *n* = 2; anterograde tracer virus *n* = 1), unilateral viral injection (hM3Dq virus *n* = 2). Therefore, 66 RXFP3‐Cre mice were included in the final data analysis. No RXFP3‐Cre‐eYFP reporter mice were excluded from the study.

### 2.2 Stereotaxic surgeries

Mice were anaesthetised under isoflurane (5% v/v in air, maintained at 0.5%–2%) and placed into a stereotaxic frame (Stoelting Co., IL, USA). Injections were performed with a Nanoject III Auto‐Nanoliter Injector (3‐000‐207; Drummond Scientific Company, PA, USA). Following infusion, the micropipette was left in situ (2 min), raised 0.1 mm, and left for a further 1 min before removal. During surgery, animals were injected with meloxicam (3 mg/kg, i.p., Troy Laboratories, Australia) and Baytril (enrofloxacin, 3 mg/kg, i.p., Bayer Australia Ltd., Australia) for analgesia and antibiotic treatment, respectively. Animals were given an additional injection of meloxicam (3 mg/kg, i.p.) 24 h post‐surgery. Animals were single‐housed and allowed to recover for 6 weeks to permit adequate viral transfection.

#### 2.2.1 Anterograde tracer surgeries

Mice were injected unilaterally with 200 nL (5 nL/s) AAV‐DJ‐hSyn‐FLEX‐mGFP‐synaptophysin‐mRuby (7.49 × 10^12^ vg/mL; Stanford University Gene Vector and Virus Core; Beier et al., 2015) targeting the lateral hypothalamus (LH)/ZI (A/P: −1.5 mm, M/L: −1.0 mm, D/V: −5.0 mm). This virus permits membrane‐bound green fluorescent protein (mGFP) expression in cell bodies and efferent fibres and red fluorescent protein (mRuby) expression at putative pre‐synaptic terminals.

#### 2.2.2 Chemogenetics surgeries

200 nL (5 nL/s) of either the Designer Receptor Exclusively Activated by Designer Drug (DREADD; AAV‐hSyn‐DIO‐hM3D(Gq)‐mCherry; 7 × 10^12^ vg/mL; Addgene, 44361‐AAV5) or control virus (AAV‐hSyn‐DIO‐mCherry; 4 × 10^12^ vg/mL; Addgene, 50459‐AAV2) were injected bilaterally in the LH/ZI (A/P: −1.5 mm, M/L: ± 1.0 mm, D/V: −5.0 mm) using methods described previously (Chen et al., 2016).

### 2.3 Chemogenetic activation of LH/ZI^RXFP3^ cells during behaviour

#### 2.3.1 Fear conditioning apparatus

Behavioural data were collected using the Contextual Near Infra‐Red Fear Conditioning System and Video Freeze® system (Med Associates, VT, USA). These experiments were conducted in standard fear conditioning chambers (Med Associates, VT, USA) as described previously (Handford et al., 2014; Short et al., 2017). A tone (volume: 80 dB; frequency: 5000 Hz, rise time 50 ms) was used as the conditioned stimulus (CS). A 1.0 mA, 1‐s footshock was used as the unconditioned stimulus (US). All sessions were recorded, and the Video Freeze® software automatically scored mouse freezing behaviour. Freezing was defined by a motion threshold of <50 pixel changes/frame for a minimum duration of 30 frames (1 s), as reported previously (Luikinga et al., 2019).

#### 2.3.2 Fear conditioning protocol

On day 1 (fear conditioning), mice were placed in the fear chamber for 1210 s in total. Baseline freezing was measured in the first 120 s, followed by nine tone‐foot shock pairings. Each tone persisted for 10 s, which co‐terminated with a 1‐s footshock. Inter‐trial intervals ranged from 85 to 135 s (mean = 110 s). Only freezing behaviours during the first 9 s of the tone were recorded to avoid confounding effects of the shock. On day 2 (fear extinction), mice were placed into the fear chamber and baseline freezing was measured in the first 120 s, after which mice were exposed to 30 CS presentations (inter‐trial intervals = 10 s) without footshock. Before extinction, all mice were injected with clozapine‐N‐oxide (CNO; Advanced Molecular Technologies, Australia; AMTA056‐CO16; 342333‐69‐7; 3 mg/kg; i.p.) and left for 30 min in their home cage before being placed into fear chambers. On day 3 (test), mice were placed into the fear chamber and baseline freezing was measured in the first 120 s, after which mice were exposed to 30 CS presentations (inter‐trial intervals = 10 s) without footshock. All mice underwent the test session treatment‐free.

#### 2.3.3 Locomotor test

Locomotor activity was assessed using open‐field chambers (Med Associates, VT, USA). On days 1 and 2 (habituation), animals were injected with saline (10 mL/kg, i.p.) 30 min before behavioural testing, and then placed into chambers for locomotor activity measurement (20 min). On day three (test), mice were injected with CNO (3 mg/kg, i.p.) 30 min before behavioural testing, and then placed into chambers for locomotor activity measurement (20 min). Movement was tracked and recorded using Activity Monitor software (Med Associates).

### 2.4 Tissue preparation and histology

Some mice that underwent behavioural testing were randomly selected for Fos analysis and received a CNO injection (3 mg/kg, i.p.) 2 h before perfusion. Mice used for immunohistochemical analysis were anaesthetised with sodium pentobarbitone (80 mg/kg, i.p., Virbac, Australia) and transcardially perfused as described previously (Charlton et al., 2019; Cullity et al., 2019). Mice used for RNAscope™ were anaesthetised with sodium pentobarbitone (80 mg/kg, i.p., Virbac, Australia); brains were immediately extracted and snap‐frozen over dry ice. Brains were sectioned at 40 μm for immunohistochemistry (IHC) and 8 μm for RNAscope™ on a Leica CM1950 Cryostat (Leica Biosystems, Germany). Sections for IHC were stored in a 1‐in‐4 series in sodium azide (0.1% w/v in 0.1 M PBS). Sections for RNAscope™ were immediately mounted onto SuperFrost® Plus slides (ThermoFisher Scientific, Australia) and then stored at −80°C until required.

### 2.5 Immunohistochemistry (IHC)

1‐in‐4 series from the rostral pole of the LH to the caudal part of the dorsal zona incerta/ventral zona incerta (ZId/ZIv; A/P: −0.34 mm to −2.80 mm) were processed for fluorescent IHC as described previously (Walker et al., 2017), with appropriate modifications to the antibodies used (Table 1). 3,3′‐Diaminobenzidine tetrahydrochloride (DAB; Sigma‐Aldrich, Australia) IHC for Fos was performed on every fourth section pre‐LH (A/P: 1.18 mm to −0.22 mm) and post‐ZId/ZIv (A/P: −2.92 mm to −4.84 mm) as described previously (Charlton et al., 2019), with appropriate modification to the antibodies used [primary: goat anti‐cFos polyclonal (1:2000; Santa Cruz Biotechnology, CA, USA, sc‐52‐G); secondary: biotinylated horse anti‐goat (1:500, Vector Laboratories, CA, USA, BA‐9500)] and omission of the cresyl violet counterstain.

**TABLE 1:** Antibodies used for fluorescent immunohistochemistry.

Antibody	Primary/secondary	Dilution	Source	Cat. No.	RRID
Rabbit anti‐DsRed polyclonal	Primary	1:1000 (mCherry) 1:2000 (tdTomato)	Takara Bio Clontech	632 496	AB_10013483
Goat anti‐cFos polyclonal	Primary	1:500	Santa Cruz Biotechnology	sc‐52‐G	AB_2629503
Chicken anti‐GFP polyclonal	Primary	1:1000	Abcam	ab13970	AB_300798
Goat anti‐ppMCH polyclonal	Primary	1:1000	Santa Cruz Biotechnology	sc‐14 509	AB_2237276
Mouse anti‐orexin‐A monoclonal	Primary	1:1000	Santa Cruz Biotechnology	sc‐80 263	AB_1126868
Sheep anti‐TH polyclonal	Primary	1:1000	Abcam	ab113	AB_297905
AF‐594‐conjugated anti‐rabbit IgG raised in donkey	Secondary	1:500	Invitrogen	A‐21207	AB_141637
AF‐488‐conjugated anti‐goat IgG raised in donkey	Secondary	1:500	Invitrogen	A‐11055	AB_2534102
AF‐488‐conjugated anti‐chicken IgG raised in donkey	Secondary	1:200	Jackson ImmunoResearch	703‐545‐155	AB_2340375
AF‐488‐conjugated anti‐mouse IgG raised in donkey	Secondary	1:200	Invitrogen	A‐21202	AB_141607
AF‐647‐conjugated anti‐goat IgG raised in donkey	Secondary	1:200	Invitrogen	A‐21447	AB_2535864

### 2.6 RNAscope™ fluorescent in situ hybridisation

RNAscope™ fluorescent in situ hybridisation (ACDBio, CA, USA) was used to detect RXFP3 (Mm‐*Rxfp3*; 439381‐C2) and Cre (312281) in the LH and ZI to validate the fidelity of the RXFP3‐Cre mouse. All incubation steps were performed in a humid environment at 40°C unless stated otherwise, as performed previously (Ch'ng et al., 2019). Slides were placed in an oven at 60°C (overnight), incubated in RNAscope™ Hydrogen Peroxide Reagent (8 min), washed with dH_2_O (2× 1 min) and air‐dried. A hydrophobic barrier was drawn around each section with an ImmEdge™ pen (Vector Laboratories, CA, USA) before protease treatment (Protease Plus, 20 min), then washed with RNAscope™ 1× Wash Buffer (2× 2 min). Sections were then incubated in Mm‐*Rxfp3* probe (ACDBio, CA, USA, 439381‐C2) diluted 1:50 in *Cre* probe (ACDBio, CA, USA, 312281; 1 h 30 min). All subsequent steps followed the manufacturer's instructions for the RNAscope™ Multiplex Fluorescent Reagent Kit v2 Assay (ACDBio, CA, USA, 323100‐USM). Briefly, sections were incubated in Amp1 (30 min), Amp2 (30 min), and Amp3 (15 min) for probe amplification. Opal 690 (1:750, Akoya Biosciences, MA, USA, FP1497001KT) and Opal 520 (1:750, Akoya Biosciences, MA, USA, FP1487001KT) were used as fluorophores to visualise RXFP3 and Cre mRNA, respectively. All reagents used were from the RNAscope™ Multiplex Fluorescent Reagent Kit v2—Mm (ACDBio, CA, USA, ADV323136) unless stated otherwise.

### 2.7 Microscopy and image acquisition

Fluorescent images for all experiments except for anterograde tracing were taken with a Zeiss Axio Imager M2 (Carl Zeiss AG, Germany) with epifluorescent Colibri 7.7 LED illumination. Stitched photomicrographs were obtained at 10× and 20× magnification with a NeoFluar 10×/0.45 (WD = 2.1 mm) lens and a Plan‐Apo 20×/0.8 (WD = 0.55 mm) lens, respectively. The Stereo Investigator (MBF Bioscience, VT, USA) imaging software was used to acquire images. For anterograde tracing analysis, fluorescent images were taken with a Zeiss Axio Imager 2 confocal laser scanning microscope (Carl Zeiss AG, Germany). Stitched photomicrographs were obtained at 20× magnification with a Plan‐Apo 20×/0.8 (WD = 0.55 mm) lens. The ZEN Black software (Carl Zeiss AG, Germany) imaging software was used to acquire images. Brightfield images were taken with a 3D Histech Panoramic SCAN II (3DHistech Ltd., Hungary). Stitched photomicrographs were acquired at 20× magnification with a Zeiss Plan‐Apo (WD = 0.55 mm) lens. The SlideCenter 3.1 software (3D Histech Ltd., Hungary) was used to view images.

### 2.8 Data analysis

Statistical analyses were performed using SPSS (IBM Corporation, NY, USA) and PRISM 7 (GraphPad Software, CA, USA). For fear extinction and test, baseline freezing was analysed by one‐way analysis of variance (ANOVA) using virus as a factor. Freezing to the CS during fear conditioning, extinction, and test were analysed by two‐way repeated measures (RM) ANOVA using virus or extinction jumping phenotype as a between‐subjects factor and CS‐US pairing as a within‐subjects factor. For all fear conditioning analyses, Mauchly's Test of Sphericity indicated that the assumption of sphericity had been violated (all *p's* < 0.001); therefore, a Greenhouse–Geisser correction was applied. For all stages of locomotor testing, data were analysed by two‐way RM ANOVA. Where significant interactions were found, post hoc Bonferroni multiple comparisons were performed where appropriate and described in the text.

For analysis of *Rxfp3* and *Cre* mRNA co‐expression in the LH/ZI, the numbers of *Rxfp3*+ cells, *Cre* + cells, and *Rxfp3*+/*Cre* + cells were quantified manually using QuPath's cell counter plugin (Bankhead et al., 2017). Individual cells were defined by the presence of a DAPI+ counterstain. DAPI+ cells were defined by QuPath's cell detection feature using the following settings: (median filter radius – 2.5 μm; sigma – 1.5 μm; minimum area – 5 μm^2^; maximum area – 400 μm^2^; threshold – 200). Cells were defined as being positive for either *Rxfp3* or *Cre* if they displayed two or more puncta within 5 μm of a DAPI‐stained cell (Ch'ng et al., 2019; Viden et al., 2021; Walker et al., 2021). Each puncta represents a single mRNA molecule (Wang et al., 2012). Counts were performed on coronal sections at four different bregma levels approximately 400 μm apart spanning the rostro‐caudal extent of the LH/ZI according to the Mouse Brain Atlas in Stereotaxic Coordinates (Paxinos & Franklin, 2004). Data are expressed as mean ± SEM.

For analysis of hM3Dq viral spread throughout the LH/ZI. The number of hM3Dq + nuclei in particular regions of the LH/ZI was quantified manually using FIJI (ImageJ, NIH, USA) software. The ZI was subdivided into a rostral sector (ZIr; Bregma −0.82 to −1.34), anterior ZId/ZIv sector (Bregma −1.46 to −1.92), and posterior ZId/ZIv sector (Bregma −2.06 to −2.54). The LH was not subdivided. Counts were performed on coronal sections 160 μm apart. A binomial logistic regression was performed to ascertain if the relative proportions of ZIr, anterior ZId/ZIv, posterior ZId/ZIv, and LH hM3Dq + counts predicted jumping vs. non‐jumping phenotype during conditioned fear extinction.

Unilateral counts of Fos + cells across the rostro‐caudal extent of each region were performed blinded to experimental group allocation using FIJI. Each area was demarcated by the Mouse Brain Atlas in Stereotaxic Coordinates mouse atlas (Paxinos & Franklin, 2004). Fos counts were analysed by independent samples *t*‐tests between hM3Dq and mCherry groups. For regional Fos counts, a minimum of 3 sections per brain region per animal was required for inclusion in the analysis. Because of technical difficulties resulting in tissue damage during processing, particular brain regions were excluded from analysis for some mice.

Inter‐regional correlations of Fos were determined by Pearson correlation analysis (Walker et al., 2019). To identify brain regions with similar patterns of coactivation for each group, hierarchical clustering analysis was performed using the calculated Pearson correlations to determine Euclidean distances between each brain region (Anversa et al., 2023; Kimbrough et al., 2020). Regions were then organised in colour‐coded matrices based on the calculated Euclidean distances using the complete linkage method to identify modules of coactivation. Hierarchical clusters were cut at 50% of the maximal height of the dendrogram. Calculation of the Euclidean distances and hierarchical clustering was performed using Python (version 3.12) in JupyterLab (version 3.4.4). Code can be found at https://github.com/BrandonKR1.

No statistical methods were employed to predetermine sample sizes; group sizes were determined based on previous studies of similar natures. For anatomical experiments, group sizes were based on Ch'ng et al. (2019). Fear conditioning, extinction, and test group sizes were based on group sizes of 10–14 (Handford et al., 2014; Luikinga et al., 2019); however, as we began to observe jumping behaviour in a subset of animals, we added additional mice. For Fos analysis, group sizes were based on Perry and McNally (2013). As the parametric tests used (*t*‐test, ANOVA) are robust to normality violations (Blanca et al., 2017; Rasch et al., 2007), no formal normality test was conducted. As per previous fear conditioning studies (Handford et al., 2014), mice that exhibited CS‐elicited freezing over 3 standard deviations from the mean would be considered outliers and excluded from analysis. However, no mice met this criterion, thus, no mice were considered outliers.

## 3 RESULTS

### 3.1 Validation of the RXFP3‐Cre mouse

**FIGURE 1:**
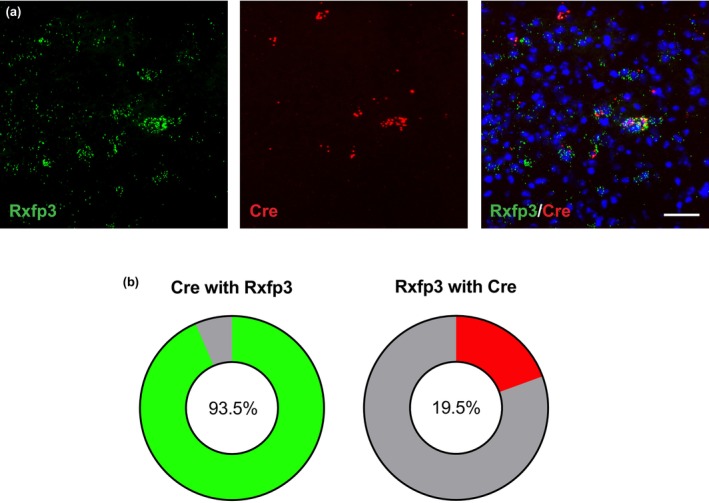
RNAscope validation of RXFP3‐Cre driver mice. (a) Representative fluorescent photomicrograph of Rxfp3 mRNA (left), Cre mRNA (middle) and merge (right) in the LH/ZI. (b) Donut graphs demonstrating the proportion of Cre‐expressing cells that express Rxfp3 and the proportion of Rxfp3 cells that co‐express Cre. Scale bar = 50 μm. *n* = 2 mice.

Validation of the RXFP3‐Cre mouse in the LH/ZI using RNAscope (Figure 1a,b) revealed that 93.5% of cells expressing Cre mRNA also co‐expressed RXFP3 mRNA in the LH/ZI of RXFP3‐Cre mice (547/585 cells), indicating high Cre specificity (Figure 1b). However, only 19.5% of RXFP3 cells expressed Cre mRNA (547/2813 cells), suggesting poor Cre penetrance (Figure 1b).

### 3.2 Efferent connectivity of LH/ZI^RXFP3^ cells

**FIGURE 2:**
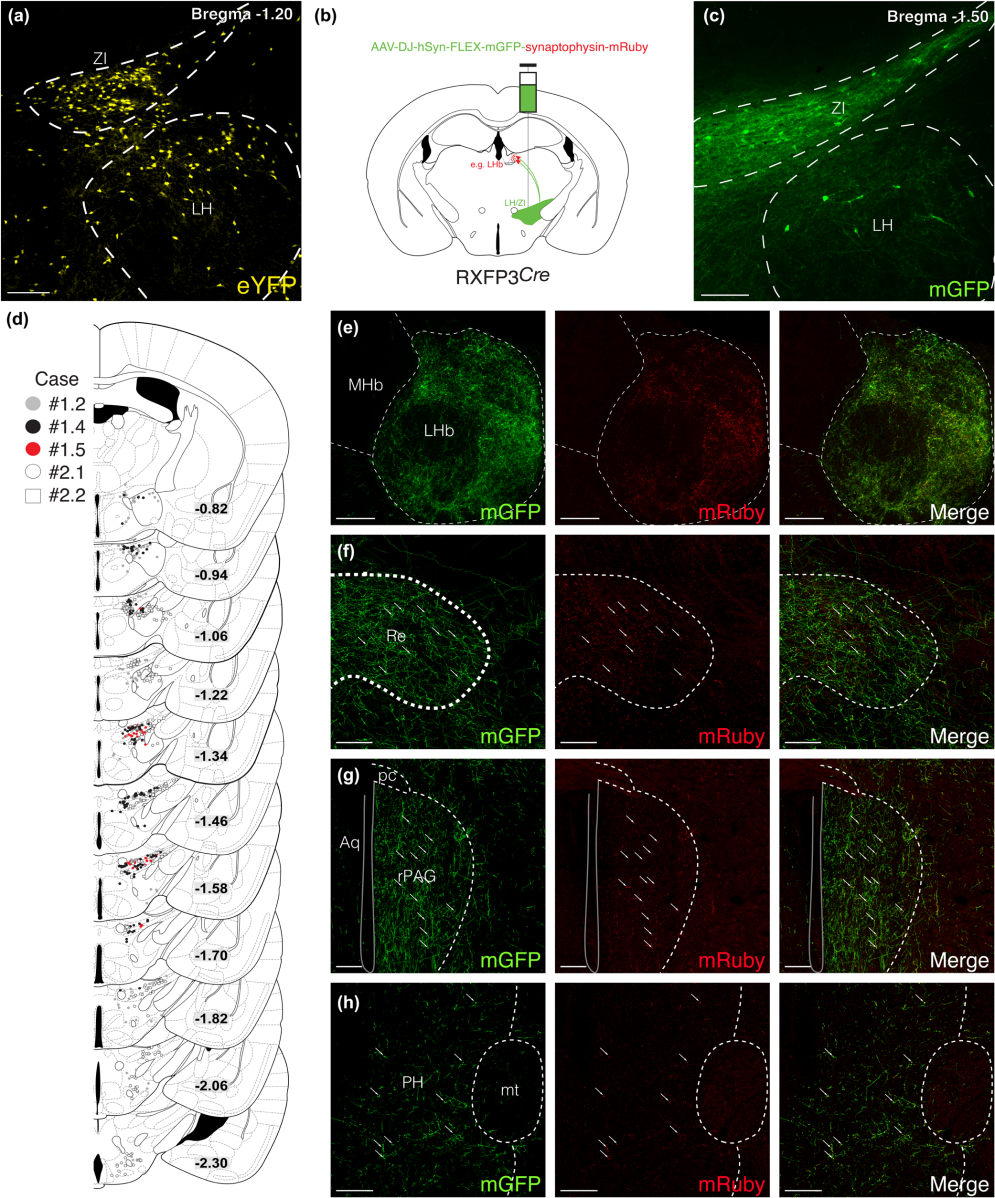
Long‐range efferent projections of LH/ZI^RXFP3^ cells. (a) Representative photomicrograph of eYFP‐expressing cell bodies in the ZI and LH of an RXFP3‐Cre‐eYFP reporter mouse (*n* = 4). (b) Diagram depicting anterograde tracing method. LH/ZI^RXFP3^ cells were targeted via unilateral stereotaxic delivery of a Cre‐dependent anterograde tracer virus (AAV‐DJ‐hSyn‐FLEX‐mGFP‐synaptophysin‐mRuby; *n* = 5) into the LH/ZI. (c) Representative fluorescent photomicrograph of anterograde tracer virus injection site in the LH/ZI. (d) Unilateral coronal diagram cascade depicting anterograde tracer injection sites across the LH/ZI. Each dot/box represents an mGFP immunoreactive cell body. Adapted from Paxinos and Franklin (2004). (e–h) Representative fluorescent photomicrographs of mGFP expression (left), mRuby expression (middle), and merge (right) of key LH/ZI^RXFP3^ efferent targets, including the lateral habenula (e), nucleus reuniens (f), rostral periaqueductal grey (g), and the posterior hypothalamus (h). Aq, cerebral aqueduct; LH, lateral hypothalamus; LHb, lateral habenula; pc, posterior commissure; PH, posterior hypothalamus; MHb, medial habenula; mt, mammillothalamic tract; Re, nucleus reuniens; rPAG, rostral periaqueductal grey; ZI, zona incerta. Scale bars = 100 μm.

In RXFP3‐Cre‐eYFP mice, eYFP+ cells were densely distributed throughout the ZI forming a population contiguous with the subincertal zone and LH (Figure 2a). Therefore, the LH was included in subsequent analyses. To map the efferent connectivity of LH/ZI RXFP3+ (LH/ZI^RXFP3^) cells, a Cre‐dependent anterograde tracer was injected into the LH/ZI of RXFP3‐Cre mice (Figure 2b). Clusters of mGFP+ immunoreactive perikarya were observed throughout the rostral and medial ZI, subincertal zone, and anterodorsal LH (Figure 2c,d). A sparse population of mGFP+ cells populated the caudal LH between the fornix and medial tuberal nucleus, only in some mice (Figure 2d). Extensive mGFP+ fibres and mRuby+ puncta (marking LH/ZI^RXFP3^ pre‐synaptic terminals) were observed throughout subcortical forebrain and midbrain areas involved in fear and arousal, with the densest mGFP/mRuby expression observed in the lateral habenula, nucleus reuniens, rostral periaqueductal grey, and posterior hypothalamus (Figure 2e–h).

**FIGURE 3:**
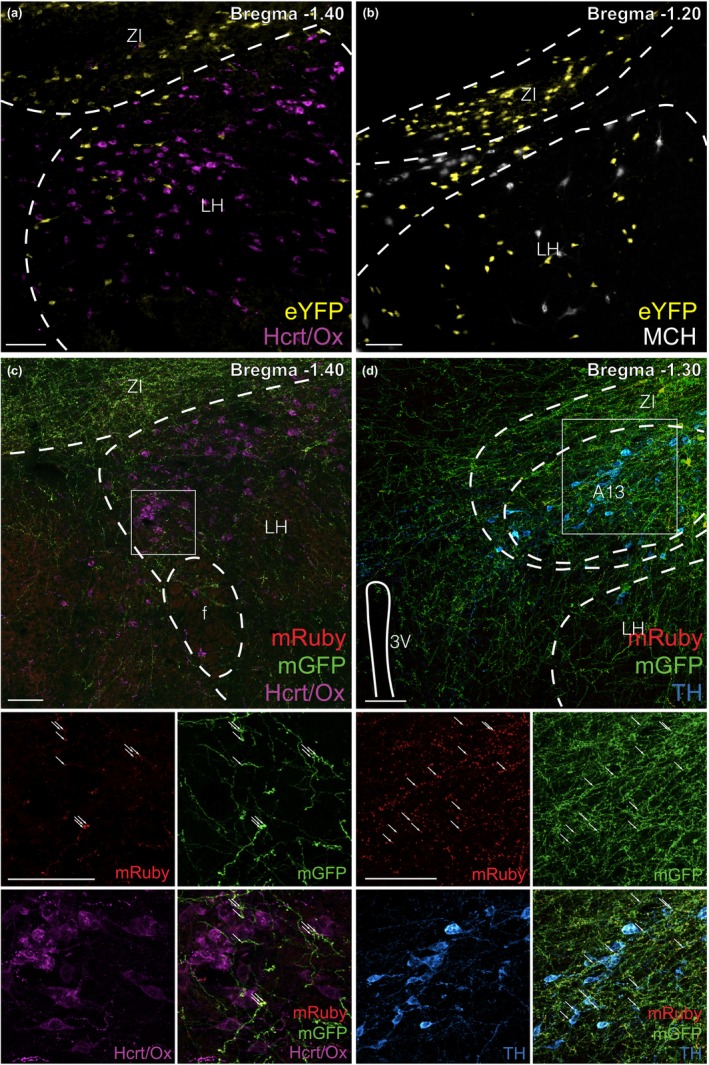
Phenotyping of LH/ZI^RXFP3^ cells and select LH/ZI^RXFP3^ efferent targets. (a, b) Representative fluorescent photomicrographs of eYFP immunoreactive neurons and hypocretin/orexin (Hcrt/Ox) (a) or melanin‐concentrating hormone (MCH) (b) immunoreactive neurons in the LH/ZI from RXFP3‐Cre‐eYFP reporter mice. (c, d) Top panels, representative fluorescent photographs of LH/ZI^RXFP3^ efferent targets expressing hypocretin/orexin in the LH (c) and tyrosine hydroxylase in the ZI A13 group (d). Bottom panels display zoomed‐in images of the inset box region split by fluorescent channel. The bottom right panels display merged images of the inset box region. f, fornix; Hcrt/Ox, hypocretin/orexin; MCH, melanin‐concentrating hormone; LH, lateral hypothalamus; TH, tyrosine hydroxylase; ZI, zona incerta; 3 V, third ventricle. Arrows indicate putative synapses onto hypocretin/orexin immunoreactive cells in (c), and onto tyrosine hydroxylase immunoreactive cells in (d). Scale bars = 100 μm.

Based on the distribution of eYFP+ cells in RXFP3‐Cre‐eYFP mice (Figure 2a), we hypothesised that LH/ZI^RXFP3^ cells may be a subset of hypocretin/orexin+ or melanin‐concentrating hormone+ neurons (Adamantidis & de Lecea, 2008). However, eYFP+ cells did not display hypocretin/orexin (Figure 3a) or melanin‐concentrating hormone immunoreactivity (Figure 3b), indicating that LH/ZI^RXFP3^ cells are not a subset of the arousal‐sensitive LH hypocretin/orexin+ and LH/ZI melanin‐concentrating hormone+ neurons. We also explored if LH/ZI^RXFP3^ cells projected to neurochemically defined LH/ZI populations, as we observed extensive interconnectivity between the LH and ZI (Figure 2c). Indeed, mRuby+ puncta closely apposed hypocretin/orexin+ immunoreactive LH cell bodies (Figure 3c) and tyrosine hydroxylase+ immunoreactive A13 ZI neurons (Figure 3d). These results suggest that LH/ZI^RXFP3^ cells may locally innervate orexinergic LH neurons and dopaminergic ZI neurons.

### 3.3 Chemogenetic activation of LH/ZI^RXFP3^ cells during conditioned fear extinction

**FIGURE 4:**
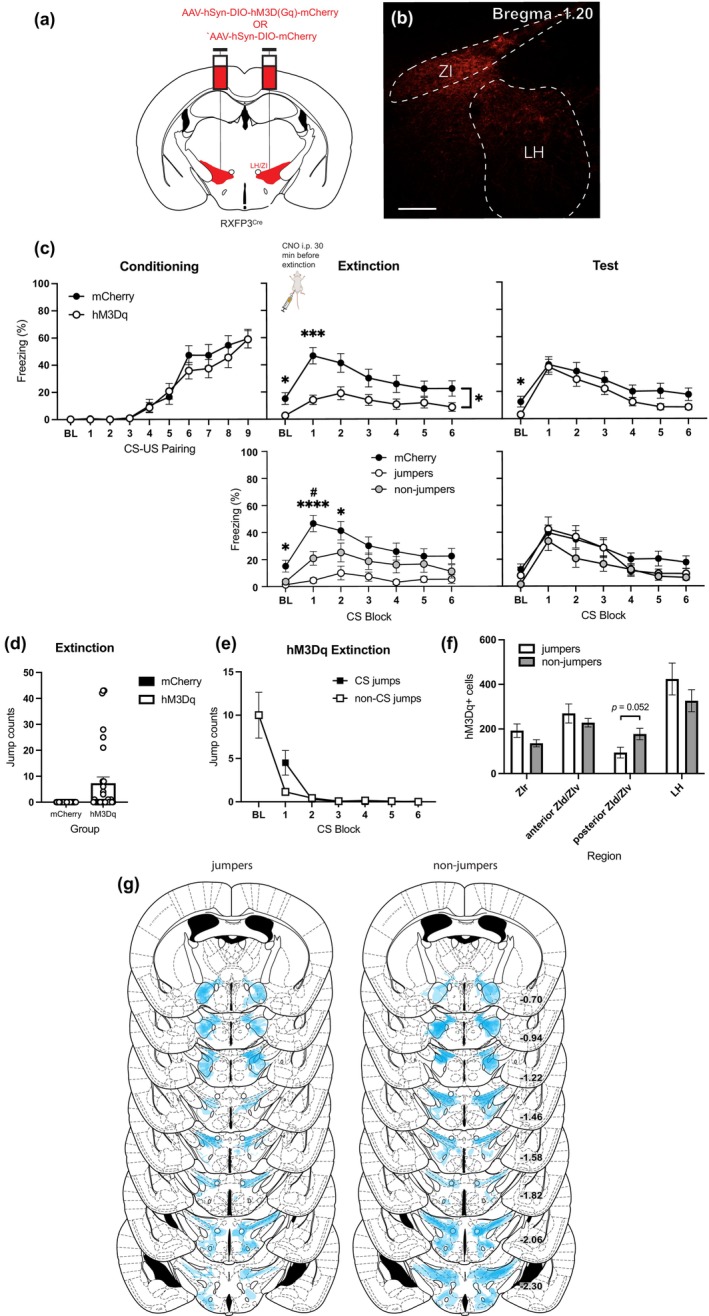
Chemogenetic activation of LH/ZI^RXFP3^ cells during context‐dependent conditioned fear extinction. (a) An excitatory DREADD (AAV‐hSyn‐DIO‐hM3D(Gq)‐mCherry) or control virus (AAV‐hSyn‐DIO‐mCherry) was bilaterally injected into the LH/ZI of RXFP3‐Cre mice to permit chemogenetic excitation of LH/ZI^RXFP3^ cells during behaviour. (b) Representative photomicrograph of hM3Dq DREADD injection site. Scale bar = 100 μm. (c) Behavioural data of freezing during fear conditioning (top left), fear extinction (top middle), and retrieval test (top right). All mice received clozapine‐N‐oxide injection (CNO; 3 mg/kg, i.p.) 30 min before the fear extinction session. Both conditioning and retrieval test days were undertaken drug‐free. Because a subset of mice displayed jumping behaviour via chemogenetic activation of LH/ZI^RXFP3^ cells during fear extinction, hM3Dq mice were subdivided into those that exhibited jumping behaviour (jumpers) and those that did not (non‐jumpers) for both extinction (bottom middle) and retrieval test (bottom right). (d) Graph showing that a subset of mice displayed jumping behaviour via chemogenetic activation of LH/ZI^RXFP3^ cells during extinction, and mCherry mice did not display jumping behaviour. (e) Jumping primarily occurred in 120‐s baseline period. Graph shows the average number of jumps during baseline and in six successive CS‐inter‐trial‐interval blocks (100 s/block). Black squares represent jumps during CS presentation (CS jumps); white squares indicate jumps during BL or the ITIs (non‐CS jumps). (f) Relative proportions of hM3Dq expression in different areas of the LH/ZI. (g) Spread of excitatory hM3Dq DREADD throughout the LH/ZI and surrounding areas for mice that displayed jumping behaviour upon chemogenetic activation during fear extinction (left, jumpers), and those that did not (right, non‐jumpers). Numbers represent the distance (mm) from Bregma. Coronal section plates adapted from Paxinos and Franklin (2004). mCherry *n* = 29, hM3Dq *n* = 32, jumpers *n* = 14, non‐jumpers *n* = 18. hM3Dq vs. mCherry, *p < 0.05, ***p < 0.001; jumpers vs. mCherry *p < 0.01, ****p < 0.0001; non‐jumpers vs. mCherry ^#^ *p* < 0.05. Data are presented as mean ± SEM. BL, baseline.

To determine if LH/ZI^RXFP3^ cells were involved in conditioned fear learning and memory, we chemogenetically activated these cells in RXFP3‐Cre mice with a Cre‐dependent hM3Dq DREADD (injected into the LH/ZI, CNO delivered i.p.; Figure 4a,b) while mice underwent conditioned fear extinction, then examined their freezing behaviour under extinction conditions drug‐free the subsequent day. During Pavlovian fear conditioning (day 1), performed without DREADD activation, mice did not freeze at baseline but increased freezing to the CS across the session (main effect of CS‐US pairing: *F*_(8,472)_ = 53.270, *p* < 0.0001; Figure 4c, left panel) independent of viral expression (main effect of virus: *F*_(1,59)_ = 0.545, *p* = 0.463). During fear extinction (day 2), activating LH/ZI^RXFP3^ cells in hM3Dq mice reduced baseline freezing compared to controls (main effect of virus: *F*_(1,59)_ = 6.668, *p* = 0.012; Figure 4c, top middle panel). CS‐elicited freezing decreased across the session (main effect of CS block: (*F*_(5,285)_ = 14.923, *p* < 0.0001)). Additionally, there was a significant main effect of virus (*F*_(1,59)_ = 6.889, *p* = 0.011) and a significant virus × CS block interaction (*F*_(5,295)_ = 4.477, *p* = 0.004). Bonferroni multiple comparisons test revealed that hM3Dq mice froze less than mCherry controls in extinction block one (*p* = 0.0002) but not in any other block (all *p*s >0.05). Some hM3Dq mice (*n* = 14) displayed jumping behaviour during fear extinction (Figure 4d; Video S1). Jump counts were not linked to CS presentation, primarily occurring during extinction baseline (Figure 4e). To determine if the behavioural phenotype observed during extinction (i.e., jumping versus non‐jumping) affected freezing, we reanalysed the extinction data with hM3Dq mice divided by phenotype. Indeed, during extinction baseline, there was a main effect of phenotype (*F*_(2,58)_ = 3.696, *p* = 0.031; Figure 4c, bottom middle panel). Bonferroni multiple comparisons test showed that jumpers displayed lower baseline freezing than mCherry controls (*p* = 0.039), but non‐jumpers did not (*p* = 0.256). During conditioned fear extinction, there was a main effect of phenotype (*F*_(2,58)_ = 5.131, *p* = 0.009) and a phenotype × CS block interaction (*F*_(10,290)_ = 2.611, *p* = 0.017).

Bonferroni multiple comparisons test indicated that jumpers displayed significantly lower freezing to the CS compared to mCherry controls during blocks 1 (*p* < 0.001) and 2 (*p* = 0.013), but not in blocks 3–6 (all *p*s >0.05). Additionally, non‐jumpers displayed significantly lower freezing to the CS compared to mCherry controls during block 1 (*p* = 0.035), but not in any other block (all *p*s >0.05). Taken together, the freezing differences observed between hM3Dq mice and controls during extinction baseline and conditioned fear extinction were primarily driven by mice that displayed jumping behaviour upon chemogenetic activation of LH/ZI^RXFP3^ cells.

Given that the ZI is a functionally heterogeneous structure and exhibits functional differences from the LH, we posited that the number of hM3Dq‐expressing cells in particular sectors of the LH/ZI may have predicted the observed jumping phenotype. As such, we performed a binomial logistic regression with jumping phenotype as the dependent variable, and hM3Dq + cell counts in the rostral ZI (ZIr), anterior ZId/ZIv, posterior ZId/ZIv, and LH as predictor variables. The regression model displayed a trend towards significance (*χ* ^2^ = 8.572, *p* = 0.073), explained 32.5% of the variance (Nagelkerke *R* ^2^) in jumping vs non‐jumping phenotype, and correctly classified 74.2% of the cases. Analysis of each individual predictive factor indicated that posterior ZId/ZIv count could predict jumping vs non‐jumping phenotype (trend, *p* = 0.052), but ZIr (*p* = 0.507), anterior ZId/ZIv (*p* = 0.589), and LH (*p* = 0.469) counts could not. Taken together, a decreasing number of hM3Dq + cells in the posterior ZId/ZIv was associated with an increased likelihood of mice displaying jumping behaviour upon chemogenetic activation of LH/ZI^RXFP3^ cells during conditioned fear extinction (Figure 4f,g).

During test (day 3), performed without DREADD activation, there was no main effect of virus on CS‐elicited freezing throughout the session (*F*_(1,59)_ = 1.347, *p* = 0.251; Figure 4c, right panel). However, there was a significant effect of virus on baseline freezing (*F*_(1,59)_ = 4.526, *p* = 0.038; Figure 4c, right panel), indicating that hM3Dq mice displayed less baseline freezing than mCherry mice. Reanalysis of the test data with hM3Dq mice divided by phenotype revealed no main effect of phenotype on baseline freezing (*F*_(2,58)_ = 2.306, *p* = 0.109) or CS‐elicited freezing (*F*_(2,58)_ = 0.798, *p* = 0.455). In summary, chemogenetically activating LH/ZI^RXFP3^ cells during extinction reduced initial baseline freezing the next day regardless of extinction jumping phenotype without affecting CS‐elicited freezing.

### 3.4 Chemogenetic activation of LH/ZI^RXFP3^ cells augments several indices of locomotor behaviour

**FIGURE 5:**
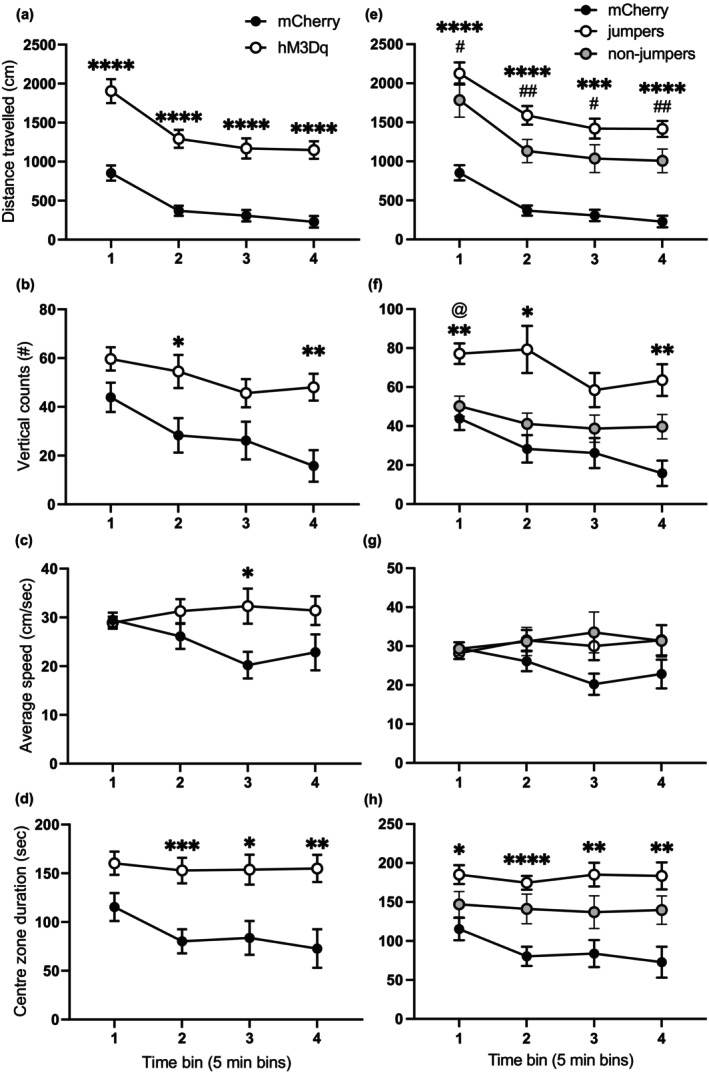
Chemogenetic activation of LH/ZI^RXFP3^ cells during an open‐field test. (a–d) Locomotor data showing (a) ambulatory distance, (b) vertical counts, (c) average ambulatory speed, (d) and centre zone duration of hM3Dq mice compared to mCherry mice. (e–h) Locomotor data showing (e) ambulatory distance, (f) vertical counts, (g) average ambulatory speed, (h) and centre zone duration with hM3Dq mice divided into those that displayed jumping behaviour upon chemogenetic activation of LH/ZI^RXFP3^ cells during fear extinction (jumpers) and those that did not (non‐jumpers). Total *n* = 44. mCherry *n* = 23, hM3Dq *n* = 21, jumpers *n* = 7, non‐jumpers *n* = 14. hM3Dq vs mCherry *p < 0.05; **p < 0.01; ***p < 0.001; ****p < 0.0001. Jumpers vs. non‐jumpers, ^@^ *p* < 0.05. Non‐jumpers vs. mCherry, ^#^ *p* < 0.05, ^##^ *p* < 0.01. Data are presented as mean ± SEM.

To investigate if LH/ZI^RXFP3^ cells affected locomotor activity, we chemogenetically activated these cells while mice were in an open field. hM3Dq mice travelled a significantly greater distance (main effect of virus: *F*_(1,40)_ = 49.56, *p* < 0.0001), had a higher vertical count (rearing; main effect of virus: *F*_(1,40)_ = 7.79, *p* = 0.008) and average ambulatory speed (main effect of virus: *F*_(1,40)_ = 4.398, *p* = 0.0423), and spent more time in the centre zone (main effect of virus: *F*_(1,40)_ = 11.81, *p* = 0.0014) compared to mCherry controls. There was a significant virus × time bin interaction for vertical counts (*F*_(3,120)_ = 3.557, *p* = 0.0164; Figure 5b), a significant virus × time bin interaction for average ambulatory speed (*F*_(3,120)_ = 3.78, *p* = 0.0123; Figure 5c) and a trend of a virus × time bin interaction for centre zone duration (*F*_(3,120)_ = 2.603, *p* = 0.0551; Figure 5d). Post hoc Bonferroni multiple comparisons revealed that hM3Dq mice travelled a significantly greater distance in all time bins (all *p*s <0.0001; Figure 5a), had increased rear counts in time bins two and four (all *p*s <0.05; Figure 5b), reduced average ambulatory speed in time bin three (*p* = 0.0442, Figure 5c), and spent significantly more time in the centre zone in time bins two to four (all *p*s <0.05; Figure 5d) compared to controls. Subdividing the hM3Dq mice by conditioned fear extinction phenotype revealed a main effect of phenotype for total ambulatory distance (*F*_(2,39)_ = 28.84, *p* < 0.0001; Figure 5e), vertical counts (*F*_(2,39)_ = 6.796, *p* = 0.0029; Figure 5f), and centre zone duration (*F*_(2,39)_ = 7.009, *p* = 0.0025; Figure 5h), but not for average ambulatory speed (*F*_(2,39)_ = 2.173, *p* = 0.1275, Figure 5g). Additionally, there was a significant phenotype × time bin interaction for vertical counts (*F*_(6,117)_ = 2.727, *p* = 0.0162) but not for all other locomotor measures (all *p*s >0.05). Post hoc Bonferroni multiple comparisons revealed that jumpers and non‐jumpers travelled a significantly greater distance than mCherry controls in all time bins (all *p*s <0.05; Figure 5e), but jumpers did not travel a significantly greater distance than non‐jumpers in any time bin (all *p*s >0.05), indicating that jumpers were not driving the increased ambulatory distance observed in the hM3Dq mice. Additionally, though jumpers had increased vertical counts and centre zone duration compared to mCherry controls in most time bins (*p*s <0.05; Figure 5f,h), jumpers did not significantly differ from non‐jumpers in these measures for every time bin except for vertical counts time bin one (*p*s >0.05; Figure 5f,h). Non‐jumpers did not significantly differ from mCherry controls in vertical counts and centre zone duration during all time bins. Taken together, activating LH/ZI^RXFP3^ cells increased several indices of locomotor behaviour. However, some of these observed increases were driven by the subset of chemogenetically activated mice that displayed jumping during conditioned fear extinction.

### 3.5 Chemogenetic activation of LH/ZI^RXFP3^ cells alters patterns of brain‐wide Fos expression

**FIGURE 6:**
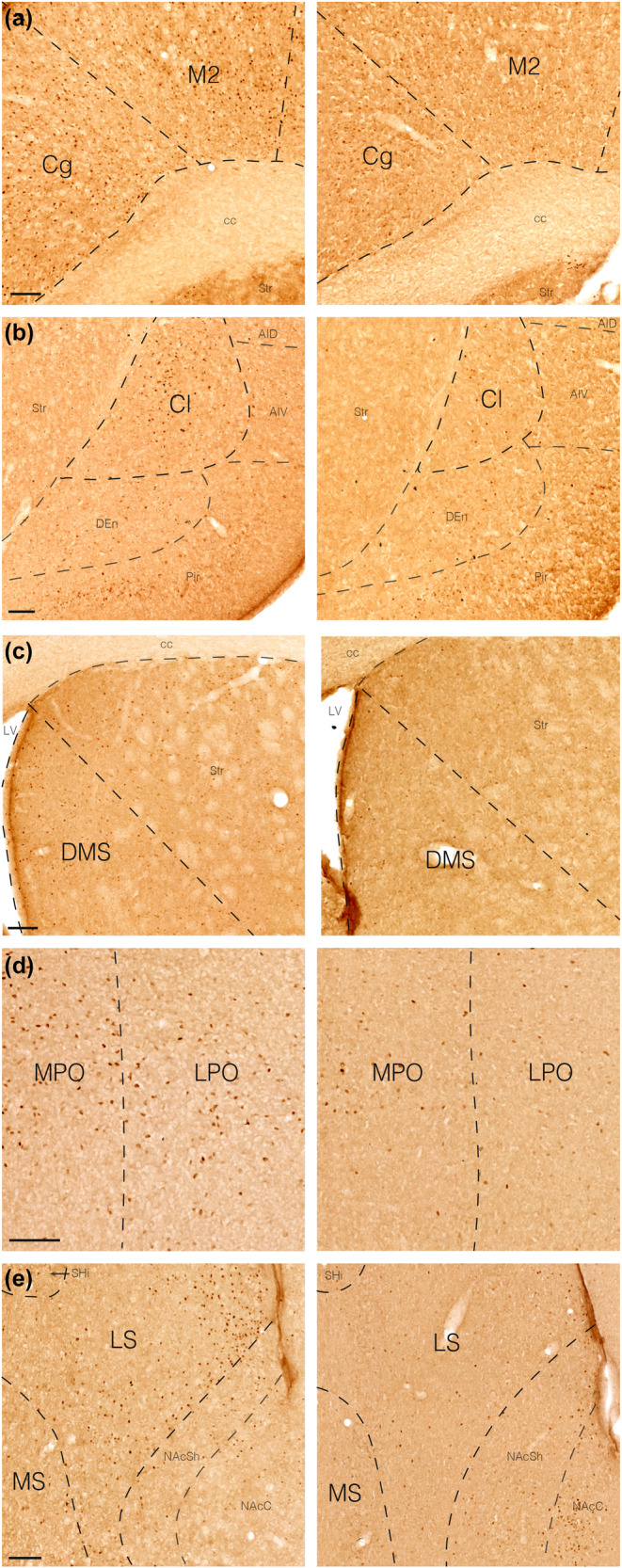
Effects of chemogenetically activating LH/ZI^RXFP3^ cells on Fos expression. (a–e) Representative brightfield photomicrographs of Fos expression in the cingulate cortex, secondary motor cortex (a), claustrum (b), dorsomedial striatum (c), medial preoptic area, lateral preoptic area (d), medial septum, and lateral septum (e) in chemogenetically activated mice (hM3Dq, left panels) versus controls (mCherry, right panels). AID, anterior insula, dorsal; AIV, anterior insula, ventral; cc, corpus callosum; Cg, cingulate cortex; Cl, claustrum; DEn, dorsal endopiriform cortex; DMS, dorsomedial striatum; LPO, lateral preoptic area; LS, lateral septum; LV, lateral ventricle; M2, secondary motor cortex; MPO, medial preoptic area; MS, medial septum; NAcC, nucleus accumbens core; NAcSh, nucleus accumbens shell; SHi, septohippocampal nucleus; Str, striatum. Scale bars = 100 μm.

Fos immunohistochemistry was employed to determine patterns of brain activity after chemogenetic activation of LH/ZI^RXFP3^ cells. A greater number of Fos immunoreactive cells were found in several cortical and subcortical regions in hM3Dq mice compared to mCherry controls (Figure 6; Table 2). Notably, areas with well‐established roles in generating locomotor activity (e.g., secondary motor cortex) and regulating fear learning (e.g., lateral septum, claustrum, cingulate cortices) displayed increased Fos immunoreactivity in hM3Dq mice compared to controls (Table 2).

**TABLE 2:** Fos‐protein expression across several brain regions in control mice (mCherry) and in mice after DREADD activation of LH/ZI^RXFP3^ cells (hM3Dq).

Region	Abbreviation	Bregma (mm)	*T*‐statistic	mCherry	hM3Dq
Dorsomedial accumbens shell	dmNAcSh	+1.42 to +0.86	*t*_(15)_ = 2.479; *p =* 0.0256*	87.6 (14.35)	139.2 (11.93)
Ventromedial accumbens shell	vmNAcSh	+1.42 to +0.86	*t*_(15)_ = 1.957; *p* = 0.0692	16.75 (4.98)	43.51 (8.44)
Lateral accumbens shell	lNAcSh	+1.42 to +0.86	*t*_(15)_ = 1.076; *p* = 0.2990	14.25 (4.53)	21.10 (3.63)
Nucleus accumbens core	NAcC	+1.42 to +0.86	*t*_(15)_ = 1.819; *p* = 0.0889	55.05 (14.17)	117.3 (20.95)
Dorsomedial striatum	DMS	+1.42 to +0.86	*t*_(16)_ = 3.293; *p* = 0.0046**	97.00 (32.47)	315.8 (43.60)
Lateral septum	LS	+1.42 to +0.62	*t*_(13)_ = 6.075; *p <* 0.0001****	174.0 (22.65)	372.9 (20.09)
Medial septum	MS	+1.18 to +0.62	*t*_(15)_ = 3.873; *p* = 0.0015**	33.04 (6.96)	71.11 (6.16)
Primary motor cortex	M1	+1.18 to +0.50	*t*_(16)_ = 2.059; *p* = 0.0561	285.9 (128.6)	680.8 (118.6)
Secondary motor cortex	M2	+1.18 to +0.50	*t*_(16)_ = 3.475; *p* = 0.0031**	232.5 (68.09)	684.7 (84.60)
Cingulate cortex	Cg	+1.18 to +0.50	*t*_(16)_ = 3.707; *p* = 0.0019**	234.1 (60.85)	492.5 (38.91)
Piriform cortex	Pir	+1.18 to +0.50	*t*_(16)_ = 1.503; *p* = 0.1522	60.67 (26.12)	105.0 (16.34)
Claustrum	Cl	+1.18 to +0.50	*t*_(16)_ = 4.521; *p* = 0.0003***	38.28 (8.87)	93.44 (7.37)
Medial preoptic area	MPO	+0.38 to −0.22	*t*_(15)_ = 4.122; *p* = 0.0009***	220.8 (22.82)	406.9 (27.18)
Lateral preoptic area	LPO	+0.38 to −0.22	*t*_(15)_ = 2.997; *p* = 0.0090**	78.40 (20.09)	175.4 (18.94)
Bed nucleus of the stria terminalis, dorsal	dBNST	+0.26 to +0.02	*t*_(16)_ = 1.071; *p* = 0.3002	73.83 (10.04)	93.63 (11.95)
Bed nucleus of the stria terminals, ventral	vBNST	+0.26 to +0.02	*t*_(16)_ = 2.533; *p* = 0.0222*	49.67 (8.21)	93.04 (11.26)
Bed nucleus of the stria terminalis, posteromedial	pmBNST	−0.10 to −0.34	*t*_(9)_ = 0.7132; *p* = 0.4938	67.38 (25.30)	86.64 (14.67)
Bed nucleus of the stria terminalis, posterolateral	plBNST	−0.10 to −0.34	*t*_(9)_ = 1.540; *p* = 0.1579	27.25 (9.65)	51.79 (10.57)
Anterior hypothalamus	AH	−0.34 to −1.06	*t*_(*12*)_ = 2.293; *p* = 0.0407*	5.17 (1.75)	19.80 (3.89)
Nucleus reuniens	Re	−0.46 to −1.34	*t*_(14)_ = 2.340; *p* = 0.0346*	3.73 (1.66)	15.85 (3.35)
Paraventricular hypothalamus	PVN	−0.58 to −1.46	*t*_(12)_ = 2.132; *p* = 0.0543	6.75 (2.33)	21.17 (4.09)
Zona incerta, rostral	ZIr	−0.82 to −1.34	*t*_(11)_ = 4.009; *p* = 0.0021**	8.90 (1.20)	39.69 (5.93)
Paraventricular thalamus	PVT	−0.94 to −1.82	*t*_(13)_ = 3.048; *p =* 0.0093**	10.50 (0.75)	40.97 (6.93)
Lateral habenula	LHb	−0.94 to −1.82	*t*_(12)_ = 2.760; *p* = 0.0173*	2.73 (0.33)	8.52 (1.53)
Medial habenula	MHb	−0.94 to −1.82	*t*_(12)_ = 0.8916; *p* = 0.3902	2.05 (0.42)	3.62 (1.27)
Central medial thalamic nucleus	CM	−0.94 to −1.82	*t*_(12)_ = 0.1035; *p* = 0.1035	1.533 (0.29)	10.74 (3.82)
Entopeduncular nucleus	EPN	−1.06 to −1.58	*t*_(12)_ = 1.039; *p* = 0.3192	0.75 (0.43)	2.35 (0.94)
Submedius thalamic nucleus	Sub	−1.22 to −1.82	*t*_(14)_ = 0.7384; *p* = 0.4725	1.00 (0.47)	1.73 (0.62)
Intermediodorsal thalamic nucleus	IMD	−1.34 to −1.82	*t*_(12)_ = 1.673; *p* = 0.1201	2.80 (1.22)	11.67 (3.83)
Lateral hypothalamus	LH	−1.22 to −2.30	*t*_(14)_ = 2.400; *p* = 0.0309*	9.49 (2.65)	29.64 (5.44)
Subincertal zone	SubI	−1.34 to −1.70	*t*_(11)_ = 2.767; *p* = 0.0183*	4.75 (0.66)	13.06 (1.93)
Dorsomedial hypothalamus	DMH	−1.34 to −2.06	*t*_(13)_ = 2.982; *p* = 0.0106*	6.93 (1.24)	36.80 (6.93)
Ventromedial hypothalamus	VMH	−1.34 to −2.06	*t*_(12)_ = 0.9341; *p* = 0.3687	5.73 (1.18)	9.70 (3.05)
Zona incerta, dorsal	ZId	−1.46 to −2.18	*t*_(12)_ = 1.961; *p* = 0.0735	5.63 (1.38)	12.75 (2.19)
Zona incerta, ventral	ZIv	−1.46 to −2.18	*t*_(12)_ = 2.592; *p* = 0.0236*	6.75 (1.76)	16.52 (2.24)
Arcuate nucleus	Arc	−1.46 to −2.18	*t*_(13)_ = 1.946; *p* = 0.0736	5.73 (1.22)	10.97 (1.78)
Dorsolateral geniculate nucleus	DLG	−1.70 to −2.54	*t*_(9)_ = 0.1728; *p* = 0.8666	1.75 (0.78)	1.57 (0.64)
Ventrolateral geniculate nucleus	VLG	−1.70 to −2.54	*t*_(9)_ = 1.012; *p* = 0.3381	1.75 (1.59)	0.57 (0.14)
Posterior hypothalamus	PH	−1.82 to −2.54	*t*_(10)_ = 1.457; *p* = 0.1759	22.00 (5.97)	52.50 (14.18)
Parafascicular thalamic nucleus	PF	−2.18 to −2.54	*t*_(9)_ = 0.7659; *p* = 0.4633	5.50 (4.14)	9.43 (3.08)
CA1/CA2 of the hippocampus	CA1/CA2	−2.30 to −2.80	*t*_(13)_ = 2.653; *p* = 0.0199*	54.72 (13.22)	141.9 (25.07)
CA3 of the hippocampus	CA3	−2.30 to −2.80	*t*_(13)_ = 1.339; *p* = 0.2035	14.50 (3.47)	25.89 (6.48)
Supramammillary nucleus	SuM	−2.80 to −3.16	*t*_(10)_ = 1.930; *p* = 0.0824	19.33 (2.74)	44.72 (7.31)
Edinger‐Westphal nucleus	EW	−2.92 to −3.40	*t*_(14)_ = 0.1138; *p* = 0.9110	9.11 (2.03)	9.47 (2.08)
Ventral tegmental area	VTA	−2.92 to −3.80	*t*_(14)_ = 1.994; *p* = 0.0660	10.06 (1.18)	22.00 (4.53)
Deep mesencephalic nucleus	DpMe	−2.92 to −4.24	*t*_(14)_ = 0.386; *p* = 0.7050	60.61 (15.72)	69.40 (14.81)
Periaqueductal grey, dorsolateral	dlPAG	−3.52 to −4.04	*t*_(13)_ = 0.4756; *p* = 0.6429	13.83 (3.29)	16.22 (3.46)
Periaqueductal grey, dorsomedial	dmPAG	−3.52 to −4.04	*t*_(13)_ = 0.4454; *p* = 0.6633	18.17 (6.41)	21.33 (3.96)
Periaqueductal grey, lateral	lPAG	−3.52 to −4.04	*t*_(13)_ = 0.5241; *p* = 0.6090	43.17 (9.23)	49.81 (8.31)
Dorsal raphe nucleus	DR	−4.04 to −4.72	*t*_(10)_ = 1.403; *p* = 0.1908	11.33 (3.26)	21.48 (5.59)
Superior colliculus	SC	−4.04 to −4.72	*t*_(10)_ = 0.3712; *p* = 0.7182	49.40 (13.39)	57.62 (15.97)
Reticulotegmental nucleus	RtTg	−4.16 to −4.60	*t*_(10)_ = 0.8905; *p* = 0.3941	15.93 (5.72)	35.81 (18.18)
Periaqueductal grey, ventrolateral	vlPAG	−4.16 to −4.84	*t*_(10)_ = 1.461; *p* = 0.1748	39.73 (7.50)	60.10 (10.40)
Median raphe nucleus	MR	−4.16 to −4.96	*t*_(10)_ = 1.858; *p* = 0.0928	4.13 (1.21)	9.81 (2.40)
Paramedian raphe nucleus	PMR	−4.16 to −4.96	*t*_(10)_ = 1.561; *p* = 0.1495	3.73 (1.74)	7.57 (1.66)

*Note*: Data presented as average number of Fos‐positive cells ± SEM. *n* = 3–10/region/group.

* *p* < 0.05;

** *p* < 0.01;

*** *p* < 0.001;

**** *p* < 0.0001.

**FIGURE 7:**
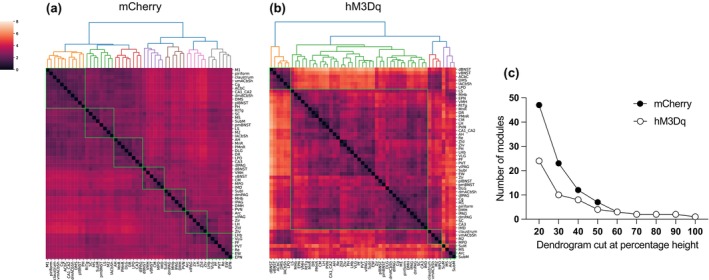
Hierarchical clustering of Euclidean distance matrices for Fos expression between hM3Dq and mCherry groups. Modules were determined by cutting each dendrogram at 50% of the maximum height and are identified by the green squares. (a, b) Euclidean distance of each analysed brain region compared to all others in control (mCherry) mice (a) or hM3Dq mice (b). Seven modules of co‐activation were identified in controls, whereas four modules of activation were identified in the hM3Dq group. (c) Number of modules for each group after cutting the dendrogram at different percentages of the tree height. Abbreviations for regions analysed can be found in Table 2. Scale bar indicates Euclidean distance.

Using hierarchical clustering analysis, several modules containing brain regions with similar coactivation patterns were identified for both the mCherry and hM3Dq groups. Coactivation patterns in the mCherry mice (Figure 7a) were organised into seven small modules, where regions within these modules were highly correlated (reflected by the darker red colours). In contrast, hM3Dq mice displayed decreased modularity compared to controls and were organised into one large and three smaller modules (Figure 7b). hM3Dq mice consistently displayed a lower number of modules compared to mCherry controls across different clustering levels (Figure 7c). Coactivation patterns in hM3Dq mice were not as highly correlated compared to mCherry controls (as reflected by the lighter orange colours). Of note, the first module in the hM3Dq mice (top left, Figure 7b) primarily consisted of subdivisions of the striatum and extended amygdala, which had largely opposing patterns of coactivation compared to the large second module (middle), which consisted primarily of limbic, hypothalamic, thalamic, and midbrain regions, including all subdivisions of the periaqueductal grey, lateral habenula, nucleus reuniens, and posterior hypothalamus. Taken together, these results suggest that activating LH/ZI^RXFP3^ cells generates distinct patterns of coactivation between numerous areas involved in stress, arousal, and defensive behaviour.

## 4 DISCUSSION

We set out to determine how a previously unstudied neuronal population contributes to conditioned fear expression. We first aimed to characterise the downstream projections of ZI RXFP3+ cells. Since we observed numerous RXFP3+ neurons in the adjacent LH, these were included in our analysis. We observed that LH/ZI^RXFP3^ cells projected to regions involved in fear learning and arousal, such as the nucleus reuniens (Ramanathan & Maren, 2019; Venkataraman et al., 2021), periaqueductal grey (McNally & Cole, 2006), and LH hypocretin/orexin+ cells (Berridge et al., 2010). To examine the functional role of these neurons, we used chemogenetics to activate LH/ZI^RXFP3^ cells in a fear conditioning protocol and an open‐field test. This activation reduced CS‐elicited freezing and induced jumping behaviour in a subset of mice. Additionally, chemogenetically activating LH/ZI^RXFP3^ cells increased locomotor activity in the open‐field, increased Fos expression in arousal and defensive behaviour‐implicated regions and generated distinct patterns of co‐activation between numerous arousal and defensive behaviour‐implicated regions. Collectively, we have demonstrated that activating LH/ZI^RXFP3^ cells augments arousal and generates brain‐wide activation patterns conducive to behavioural activation.

Given that relaxin‐3/RXFP3 signalling affects arousal, we predicted that activating LH/ZI^RXFP3^ cells during extinction would affect conditioned fear expression. Though we observed reduced conditioned freezing at the beginning of extinction, we also observed increased general locomotion in the open field test following activation of LH/ZI^RXFP3^ neurons. Although this may present a confound to the decreased freezing, a subset of chemogenetically activated mice displayed stereotypic jumping during fear extinction. This behaviour is typically indicative of escape (Wang et al., 2021), suggesting that activating these cells may not have inhibited conditioned fear expression but instead induced a change in defence strategy. Indeed, as flight is generally favoured to freezing in response to a more imminent threat (De Franceschi et al., 2016), activating LH/ZI^RXFP3^ neurons in these mice may have generated a fear response appropriate to a more dangerous situation. Given that activating LH/ZI^RXFP3^ cells induced jumping behaviour in only a subset of mice, it is likely that these cells do not comprise a single functional population. Indeed, it is not known if the LH/ZI^RXFP3^ population reflects an inhibitory phenotype, excitatory phenotype, or combination of both, which is of future interest. Additionally, as validation of the RXFP3‐Cre mouse revealed that only 19.5% of Rxfp3‐expressing cells in the LH/ZI expressed Cre, it is essential to note that the behavioural results observed were based on activating only a subset of LH/ZI^RXFP3^ cells.

Jumpers had fewer chemogenetically activated cells in the posterior ZId/ZIv compared to non‐jumpers, though the proportions of chemogenetically activated cells in the ZIr, anterior ZId/ZIv and LH were not significantly different between jumpers and non‐jumpers. Therefore, it may be that activating more RXFP3+ cells in the posterior ZId/ZIv competes with or inhibits activated populations elsewhere in the LH/ZI to suppress an overt jumping phenotype. Local inhibition would not be unprecedented given that the majority of the ZI is GABAergic, and we (and others; Mitrofanis, 2005) have observed vast interconnectivity within the ZI. Given that activating parvalbumin‐expressing neurons in this area have been shown to increase defensive behaviours induced by noxious stimuli (Wang et al., 2020), the RXFP3+ expressing cells here may reflect a population with a different neurochemical phenotype. Future studies should investigate if topologically segregated subpopulations of LH/ZI^RXFP3^ cells differ in their hodology, neurochemical composition, and functional output during defence and arousal behavioural assays.

Although our data suggest that activating LH/ZI^RXFP3^ cells primarily affected locomotion and jumping behaviour, we also observed an effect on fear extinction memory. Decreased baseline freezing during test (conducted drug‐free) was observed in mice that received DREADD activation the previous day compared to controls. However, no freezing differences were observed during test upon CS presentation. This may suggest that activating LH/ZI^RXFP3^ cells the previous day improved contextual fear extinction memory without affecting cued fear extinction memory; however, a more appropriate contextual fear conditioning paradigm that dissociates contextual fear from cued fear may be necessary to validate this finding further.

It is possible that some LH/ZI^RXFP3^ cells may have regulated conditioned fear expression but were overridden by activating antagonistic networks. For example, RXFP3+ ZI neurons may overlap with nucleus reuniens projecting GABAergic ZId/ZIv cells that enhance fear extinction recall (Venkataraman et al., 2021) as we observed dense LH/ZI^RXFP3^ efferent terminals in the nucleus reuniens and RXFP3+ cells throughout the ZId/ZIv. However, chemogenetically activating LH/ZI^RXFP3^ cells increased nucleus reuniens Fos expression, which is contradictory. This could be because LH/ZI^RXFP3^ cells also putatively synapse with glutamatergic (Henny et al., 2010; Schone et al., 2012; Torrealba et al., 2003) LH hypocretin/orexin+ cells that project to the nucleus reuniens (Peyron et al., 1998). Therefore, activating excitatory LH/ZI^RXFP3^ cells projecting to the LH hypocretin/orexin+ population may have increased nucleus reuniens activity, predominating over putative GABAergic ZId/ZIv input. Additionally, several learning and memory‐implicated regions displayed increased Fos expression upon LH/ZI^RXFP3^ cell activation, such as CA1/CA2, the cingulate cortices, and lateral septum (Bian et al., 2019; Opalka & Wang, 2020; Opitz, 2014; Ortiz et al., 2019; Rolls, 2019). However, control animals generally displayed coordinated activity of telencephalic regions concerned with cognition, while hM3Dq mice did not. This may align with the observation of disrupted frontal connectivity during acute stress (Arnsten, 2015). Additionally, several diencephalic defence and stress areas displayed higher Fos activity upon activation of LH/ZI^RXFP3^ cells, such as the dorsomedial hypothalamus, paraventricular nucleus of the hypothalamus, and paraventricular nucleus of the thalamus (Daviu et al., 2020; Ma et al., 2021; Ullah et al., 2015). However, given that chemogenetically activated mice exhibited variable hM3Dq spread, it is possible that Fos expression may differ based on topological site of chemogenetic activation. Therefore, interpretation of Fos expression should be approached with caution. Nevertheless, it is possible that activating a subset of LH/ZI^RXFP3^ cells engaged circuits responsible for modulating conditioned fear expression, but observable effects were masked by a dominant subpopulation responsible for augmenting locomotor behaviour and arousal. Future studies involving projection‐specific LH/ZI^RXFP3^ manipulations (e.g., to the nucleus reuniens) are necessary to investigate how particular subpopulations participate in conditioned fear expression.

The possibility remains that increased locomotion and jumping may have reflected a general increase in motor output, especially given that LH/ZI^RXFP3^ cells putatively synapse with the dopaminergic A13 population. In future studies, trans‐synaptic retrograde viral tracing should be conducted to validate the putative connectivity between LH/ZI^RXFP3^ cells and the dopaminergic A13 population. A13 cells project to mesencephalic locomotor region nuclei (Sharma et al., 2018), an area where electrical stimulation incites strong locomotor responses (Milner & Mogenson, 1988). A recent finding has also implicated dopaminergic A13 cells in mediating prehensile movements without affecting motivation (Garau et al., 2023), suggesting that activating RXFP3^LH/ZI^ cells in this study may have increased limb movements not associated with emotional valence. Additionally, increased Fos expression was observed in motor‐related areas upon cell activation compared to controls, such as the lateral preoptic area (Subramanian et al., 2018). Notably, substantial Fos increases were observed in the secondary motor cortex and dorsomedial striatum, and optogenetic stimulation of a glutamatergic secondary motor cortex to dorsomedial striatum projection augments locomotion (Magno et al., 2019).

The increased rearing and centre zone duration observed in the open‐field test is challenging to interpret. Although rearing can be indicative of increased exploratory drive (Lever et al., 2006), it has been suggested that rearing maps onto an inverted‐U curve of stress, such that increasing stress may either augment or inhibit the behaviour dependent on baseline stress level (Sturman et al., 2018). Therefore, activating LH/ZI^RXFP3^ cells may have increased rearing behaviour due to hypervigilance rather than increased exploratory drive. Indeed, several studies have reported increased rearing behaviour upon exposure to various stressors, including cat odour (McGregor et al., 2004) and restraint stress (Zimprich et al., 2014). Although not discerned in the current study, wall‐supported rearing may indicate failed escape (Lever et al., 2006). This is consistent with increased Fos expression observed in the dorsomedial hypothalamus, as pharmacological excitation of the medial hypothalamus enhances locomotor behaviour and rearing and induces defensive jumping (Silveira & Graeff, 1992). Interestingly, the heightened centre‐zone exploration observed is typically indicative of low anxiety and can be induced by anxiolytics such as diazepam (Behlke et al., 2016). However, it has been suggested that increased centre zone exploration is indicative of anxiolysis only without a simultaneous increase in overall locomotion and rearing (Prut & Belzung, 2003). As all three indices were increased in the current study, this result may reflect increased arousal rather than anxiolysis.

It is important to highlight that validation of the RXFP3‐Cre mouse line using RNAscope revealed that the penetrance of Cre expression in RXFP3+ cells was relatively poor. Therefore, our viral tracing and behavioural data likely under‐represent the efferent connectivity and functional outputs of the entire LH/ZI^RXFP3^ population. However, Rxfp3+ LH/ZI cells that did not express Cre were not biased to anatomically distinct LH/ZI locations, suggesting that our Cre vectors at least captured an anatomically representative subset of LH/ZI^RXFP3^ cells. Future studies might further seek to determine whether our Cre vectors captured a neurochemically biased sample of the available LH/ZI^RXFP3^ population. However, the lack of anatomical specificity of Cre expression suggests this is unlikely. Indeed, a putative explanation for this is that the RXFP3‐Cre transgene was silenced stochastically in some LH/ZI^RXFP3^ cells. The bacterial artificial chromosome (BAC) RXFP3‐Cre construct integrates randomly into the genome; however, the copy number can vary (Schmidt et al., 2013; Song & Palmiter, 2018; Yang & Gong, 2005). Therefore, one of these transgenes may integrate into a heterochromatic site or another transcriptionally suppressed region (Chang et al., 2013), causing Cre silencing for a subset of RXFP3‐expressing cells. Alternatively, as only a small amount of Cre is necessary to drive successful gene recombination (Orban et al., 1992), it is possible that the RNAscope method failed to detect low levels of Cre transcript in some RXFP3+ cells. This is probable given that RXFP3‐Cre‐eYFP reporter mice displayed a similar pattern of eYFP expression to Rxfp3 mRNA expression in our RNAscope experiments. Therefore, although Cre mRNA was undetectable in some LH/ZI^RXFP3^ cells in RXFP3‐Cre mice, Cre must have been present in many of these cells to permit eYFP fluorophore expression in RXFP3‐Cre‐eYFP mice. To confirm this, a future study should be conducted that examines Rxfp3 mRNA with eYFP fluorophore expression in the LH/ZI. Furthermore, the behavioural effects observed in this study were generated by Cre‐dependent viral vectors and thus would be reflective of Cre‐expressing neurons in the LH/ZI. Given that the majority (93.5%) of Cre‐expressing neurons in the LH/ZI expressed RXFP3, any behavioural effects observed in our experiment can confidently be attributed to the activation of LH/ZI^RXFP3^ neurons.

In summary, we have provided an initial account of the efferent connectivity and function of LH/ZI^RXFP3^ cells, primarily implicating the population in augmenting arousal. Although our neuroanatomical findings strongly suggested that activating these cells would also affect conditioned fear expression, we hypothesise this was not observed due to the gross activation of antagonistic networks in the LH/ZI. This hypothesis is consistent with the observed jumping behaviour in only a subset of chemogenetically activated animals. Moving forward, parsing out the function of discrete LH/ZI^RXFP3^ populations based on neurochemistry and connectivity is required to understand how these cells integrate into stress and arousal circuits relevant to fear‐related disorders.

## AUTHOR CONTRIBUTIONS

**Brandon K. Richards:** Investigation; writing – original draft; writing – review and editing; formal analysis. **Sarah S. Ch'ng:** Conceptualization; writing – review and editing; methodology. **Ariel B. Simon:** Investigation; writing – review and editing. **Terence Y. Pang:** Writing – review and editing. **Jee Hyun Kim:** Writing – review and editing; conceptualization. **Andrew J. Lawrence:** Conceptualization; funding acquisition; writing – review and editing; methodology; supervision. **Christina J. Perry:** Conceptualization; methodology; investigation; formal analysis; funding acquisition; supervision; writing – review and editing.

## CONFLICT OF INTEREST STATEMENT

Andrew J. Lawrence is the current Editor‐in‐Chief of the *Journal of Neurochemistry*. The authors declare no competing financial interests.

### PEER REVIEW

The peer review history for this article is available at https://www.webofscience.com/api/gateway/wos/peer‐review/10.1111/jnc.16217.

## Supporting information


Video S1.

## Data Availability

The data that support the findings of this study are available from the corresponding author upon reasonable request.
